# Supplementary material for: Identification of photoperiod-induced specific miRNAs in the adrenal glands of Sunite sheep (Ovis aries)
Source: Front Vet Sci. 2022 Jul 22;9:888207. doi: 10.3389/fvets.2022.888207 (PMC9354845; doi:10.3389/fvets.2022.888207)
Supplement: Supplementary file 1 [file Data_Sheet_1.ZIP › Supplementary file/miRDeep2 file.zip!/miRDeep2 file/Novel_Structure/SPLP42A2/Novel_291.pdf]

Provisional ID	:	NC_019478.2_36906
Score total	:	0
Score for star read(s)	:	-1.3
Score for read counts	:	0
Score for mfe	:	-0.3
Score for randfold	:	1.6
Score for cons. seed	:	
Total read count	:	212
Mature read count	:	212
Loop read count	:	0
Star read count	:	0

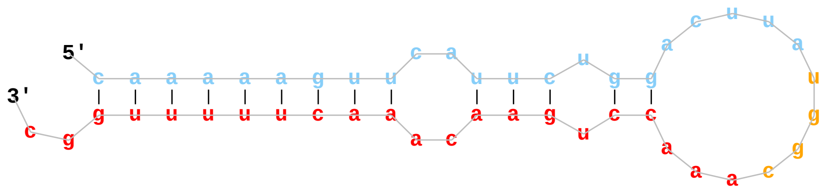

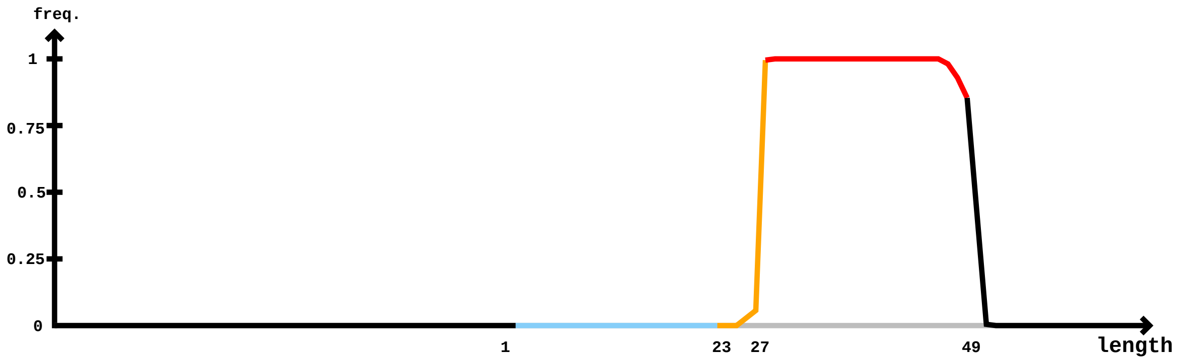

Star Mature

```
5'- uguugugagaaaaccaccaccuguuuuguaaaaguaucaggauggccaaaaaguucauucuggacuuauggcaaaccugaacaaacuuuuuggccaacucaaaaguuuuuug  -3'   exp
    .......(((((((.....((((...............)))).(((((((((((((..(((.((............)).)))..))))))))))))).......))))))).   reads   mm   sample
    ......................................................................gGaaaccugaacaaacuuuuu.....................   1       1    seq
    ......................................................................gAaaaccugaacaaacuuuuug....................   2       1    seq
    ......................................................................gcaaaccugaacaaacuuuuug....................   3       0    seq
    .......................................................................Aaaaccugaacaaacuuuuu.....................   2       1    seq
    .......................................................................Aaaaccugaacaaacuuuuug....................   2       1    seq
    .......................................................................Aaaaccugaacaaacuuuuugg...................   2       1    seq
    ........................................................................aaaUcugaacaaacuuuuu.....................   1       1    seq
    ........................................................................aaaccugaGcaaacuuuuug....................   1       1    seq
    ........................................................................aaaUcugaacaaacuuuuug....................   3       1    seq
    ........................................................................aaaUcugaacaaacuuuuugg...................   13      1    seq
    ........................................................................aaaccugaaUaaacuuuuugg...................   1       1    seq
    ........................................................................aaaUcugaacaaacuuuuuggc..................   177     1    seq
    ........................................................................aaacUugaacaaacuuuuuggc..................   1       1    seq
    ........................................................................aaaccugaacaaacuuuuuggU..................   1       1    seq
    ........................................................................Gaaccugaacaaacuuuuuggc..................   1       1    seq
    .........................................................................aaccugaacGaacuuuuuggcca................   1       1    seq
```
